# Malignant melanoma and bone resorption

**DOI:** 10.1038/sj.bjc.6603103

**Published:** 2006-04-25

**Authors:** Y S Lau, A Sabokbar, H Giele, V Cerundolo, W Hofstetter, N A Athanasou

**Affiliations:** 1 Nuffield Department of Orthopaedic Surgery, University of Oxford, Nuffield Orthopaedic Centre, Oxford OX3 7LD, UK; 2 Department of Plastic and Reconstructive Surgery, Radcliffe Infirmary, Oxford OX2 6HE, UK; 3 Weatherall Institute of Molecular Medicine, John Radcliffe Hospital, Oxford OX3 9DS, UK;; 4 Group for Bone Biology, Department of Clinical Research, University of Bern, Murtenstrasse 35, CH-3010 Bern, Switzerland

**Keywords:** melanoma, osteoclast, bone resorption, RANKL

## Abstract

The cellular and humoral mechanisms accounting for osteolysis in skeletal metastases of malignant melanoma are uncertain. Osteoclasts, the specialised multinucleated cells that carry out bone resorption, are derived from monocyte/macrophage precursors. We isolated tumour-associated macrophages (TAMs) from metastatic (lymph node/skin) melanomas and cultured them in the presence and absence of osteoclastogenic cytokines and growth factors. The effect of tumour-derived fibroblasts and melanoma cells on osteoclast formation and resorption was also analysed. Melanoma TAMs (CD14+/CD51−) differentiated into osteoclasts (CD14−/CD51+) in the presence of receptor activator for nuclear factor *κ*B ligand (RANKL) and macrophage-colony stimulating factor. Tumour-associated macrophage-osteoclast differentiation also occurred via a RANKL-independent pathway when TAMs were cultured with tumour necrosis factor-*α* and interleukin (IL)-1*α*. RT–PCR showed that fibroblasts isolated from metastatic melanomas expressed RANKL messenger RNA and the conditioned medium of cultured melanoma fibroblasts was found to be capable of inducing osteoclast formation in the absence of RANKL; this effect was inhibited by the addition of osteoprotegerin (OPG). We also found that cultured human *SK-Mel-29* melanoma cells produce a soluble factor that induces osteoclast differentiation; this effect was not inhibited by OPG. Our findings indicate that TAMs in metastatic melanomas can differentiate into osteoclasts and that melanoma fibroblasts and melanoma tumour cells can induce osteoclast formation by RANKL-dependent and RANKL-independent mechanisms, respectively.

In the past decade, there has been a substantial increase in the incidence of cutaneous melanoma in the Caucasian population; the incidence of melanoma is increasing faster than any other cancer and the number of deaths from melanoma has increased throughout the world. Osseous metastases from melanoma are more common than is generally recognised; most occur in the axial skeleton and cause bone pain, neurological symptoms and pathological fractures (DeBoer *et al*, 1996; Collaborative Ocular Melanoma Study Group, 2001).

Metastatic lesions from melanoma are usually osteolytic. The cellular and molecular mechanisms underlying this tumour osteolysis are poorly understood. Tumour cells are incapable of lacunar bone resorption and tumour osteolysis is produced by osteoclasts, multinucleated cells, which are specialised to carry out lacunar bone resorption (Mundy, 1991; Clohisy *et al*, 2000). Osteoclasts are part of the mononuclear phagocyte system, and are formed by fusion of mononuclear precursors of haematopoietic origin (Blair and Athanasou, 2004). Mononuclear osteoclast precursors circulate in the monocyte fraction and express a monocyte/macrophage antigenic phenotype (Fujikawa *et al*, 1996; Blair and Athanasou, 2004). Osteoclast differentiation from these mononuclear precursors requires the presence of macrophage-colony stimulating factor (M-CSF) and involves a receptor–ligand interaction between osteoclast precursors, which express the receptor activator for nuclear factor *κ*B (RANK), and cells in bone (e.g. osteoblasts) which express its ligand (RANKL); this process is inhibited by osteoprotegerin (OPG), which is produced by osteoblasts and other cell types (Simonet *et al*, 1997; Yasuda *et al*, 1998; Hofbauer *et al*, 2000). In addition to this RANKL-dependent mechanism of osteoclast formation, it has been shown that several cytokines and growth factors, including tumour necrosis factor-*α* (TNF-*α*), interleukin 6 (IL-6), interleukin 8 (IL-8) and transforming growth factor-*β* (TGF-*β*), can induce osteoclast formation from marrow and circulating osteoclast precursors by a RANKL-independent mechanism (Kobayashi *et al*, 2000; Kudo *et al*, 2002; Bendre *et al*, 2003; Kudo *et al*, 2003; Itonaga *et al*, 2004).

A prominent macrophage infiltrate is commonly found in many primary and secondary cancers (Bugelski *et al*, 1987; Van Ravenswaay Classen *et al*, 1992). It has been shown that tumour-associated macrophages (TAMs) are capable of osteoclast differentiation when these mononuclear phagocytes are co-cultured with bone-derived stromal cells (Quinn *et al*, 1998). In this study, our aim has been to determine the cellular mechanisms of tumour osteolysis associated with metastatic melanoma. We have examined whether TAMs in secondary melanomas are capable of differentiating into osteoclasts and determined whether this occurs by a RANKL-dependent or RANKL-independent mechanism. Melanoma cells, tumour fibroblasts and bone cells are present at sites of skeletal metastasis of a melanoma, and we have therefore also sought to analyse the manner in which these cells might influence osteoclast formation and resorption.

## MATERIALS AND METHODS

This study was approved by the Oxford Clinical Research Ethics Committee. Alpha minimum essential medium (MEM) and heat-inactivated foetal bovine serum (FBS) were purchased from Gibco Laboratories (Paisley, UK). Minimum essential medium containing 10% FBS, 100 U ml^−1^ penicillin and 10 *μ*g ml^−1^ streptomycin (MEM/FBS) was used for all cell culture experiments unless otherwise specified. Macrophage-colony stimulating factor, TNF-*α*, OPG, RANK:Fc and antibodies to TNF-*α* (TNFSF1A), TGF-*β* RII, IL-8 (CXCL8) and gp130 were obtained from R&D Systems Europe (Abingdon, UK). Soluble RANKL was obtained from Peprotech (London, UK). The human melanoma cell line *SK-Mel-29* (Ogg *et al*, 2000) was kindly provided by Professor V Cerundolo (Weatherall Institute of Molecular Medicine, Oxford, UK). All reagents used in reverse transcription and DNA amplification were obtained from Invitrogen (Paisley, UK). All experiments were incubated at 37°C in a humidified atmosphere of 5% CO_2_ and 95% air, and carried out in triplicate.

### TAMs: Isolation and culture

Tissue from six cases of secondary melanoma (five cutaneous and one lymph node metastasis) was obtained at the time of surgery at the Radcliffe Infirmary, Oxford. The five male and one female patients, all Caucasians, were aged between 57 and 83 years, and all had Stage 3 disease or more at the time of surgery.

The tumour tissue was washed in sterile phosphate-buffered saline. Fragments of the tumour were then placed in 1 mg ml^−1^ of collagenase Type 1 (Sigma-Aldrich, Dorset, UK) and incubated for 1 h. The digested tissue suspension was passed through a Falcon® 70 *μ*m pore size cell strainer (Becton Dickinson, Oxford, UK). The filtrate was centrifuged at 1800 **g** for 10 min and the cell pellet suspended in 2 ml of MEM/FBS. The cell yield was counted in a haemocytometer after lysis of red blood cells with 5% (v v^−1^) acetic acid. 1 × 10^5^ cells per well were added to dentine slices and glass coverslips in a 96-well tissue culture plate. After 3-h incubation, the dentine slices and glass coverslips were washed in MEM/FBS to remove any nonadherent cells and were then transferred into 24-well tissue culture plates containing 1 ml of MEM/FBS supplemented with one of the following factors:

1. No added factors (negative control).
2. M-CSF (25 ng ml^−1^).
3. M-CSF (25 ng ml^−1^) and RANKL (30 ng ml^−1^).
4. M-CSF (25 ng ml^−1^), TNF-*α* (20 *μ*g ml^−1^, day 4–21 of culture) and IL-1*α* (10 ng ml^−1^, day 11–21 of culture).

Cultures on coverslips and dentine slices were maintained for 14 and 21 days, respectively, with culture medium and factors replenished every 3–4 days.

### Isolation of human peripheral blood mononuclear cells (PBMCs)

Human monocytes were obtained by density gradient centrifugation of 50 ml of buffy coat cell preparation provided by the National Blood Transfusion Service (Bristol, UK). The buffy coat preparation was mixed with an equal volume of MEM and purified over Histopaque (Sigma-Aldrich, Dorset, UK). After centrifugation at 2250 **g** for 25 min, the cell layer above the Histopaque was collected, suspended in MEM, and centrifuged at 1800 **g** for 10 min. The cell pellet was resuspended in MEM and centrifuged again. 5 ml of MEM/FBS was then added to the cell pellet and the number of cells counted in a haematocytometer following lysis of red blood cells with 5% (v v^−1^) acetic acid. 5 × 10^5^ cells per well were plated immediately onto dentine slices and glass coverslips in 96-well tissue culture plates with 100 *μ*l per well of MEM/FBS. After 3-h incubation, the dentine slices and glass coverslips were washed in MEM/FBS to remove any nonadherent cells, and transferred into 24-well tissue culture plates containing 1 ml of MEM/FBS and M-CSF (25 ng ml^−1^). Positive controls were set up in the presence of M-CSF (25 ng ml^−1^) and soluble RANKL (30 ng ml^−1^).

### Cytochemical and functional assessment of osteoclast formation

Histochemistry for the expression of the osteoclast-associated enzyme, tartrate-resistant acid phosphatase (TRAP), was carried out on cell cultures on coverslips using a commercially available kit (Sigma-Aldrich, Dorset, UK). Cell preparations were fixed in citrate/acetone solution and stained for acid phosphatase, using naphthol AS-BI phosphate as a substrate, in the presence of 1.0 M tartrate (Minkin, 1982). The product was reacted with Fast Garnet GBC salt and counterstained with haematoxylin. Cell cultures on glass coverslips were also stained immunohistochemically using an indirect immunoperoxidase technique with 23C6 (Serotec, Oxford, UK) and GSR1 (Dakopatts, Glostrup, Denmark) mouse monoclonal antibodies directed against the vitronectin receptor (VNR) (Davies *et al*, 1989; Athanasou and Quinn, 1990) and CD68 (macrophage and osteoclast marker), respectively. Antibodies directed against the melanoma markers, HMB-45 and S100 (Dakopatts, Glostrup, Denmark) were also employed for phenotypic characterisation.

Functional evidence of TAM-osteoclast formation was determined by a lacunar resorption assay system using cell cultures on dentine slices (Quinn *et al*, 1998). After 21 days incubation, the cells were removed from the dentine slices by treatment with 1 M ammonium hydroxide. The dentine slices were washed in distilled water and ultrasonicated to remove adherent cells, then stained with 0.5% (w v^−1^) toluidine blue to reveal areas of lacunar resorption and examined by light microscopy.

### Melanoma-derived fibroblasts: Isolation and culture

After enzyme digestion of the tumour, the cell pellet obtained was resuspended in MEM/FBS and placed in 25 cm^2^ tissue culture flasks. The medium was changed after 24-h incubation and then at 5–7 day intervals. These cultures, containing spindle-shaped fibroblast-like cells, were passaged when confluent and re-cultured twice. After passage 2, the cells were cultured for a further 2–3 weeks until confluent; conditioned medium (CM) was then collected after a further 48-h incubation.

### Melanoma-derived fibroblasts: total RNA extraction and RT–PCR

For RT–PCR studies, the fibroblasts were removed by treatment with trypsin (0.25%)/EDTA (1 mM) solution. Total RNA extraction was carried out using the RNeasy® mini kit (QIAGEN, Hombrechtikon, Switzerland), according to the manufacturer's instructions. Single-strand complementary DNA (cDNA) was synthesised from 2.0 *μ*g of total RNA according to standard protocols using the SuperScript® First-Strand Synthesis System for RT—PCR. Complementary DNA was amplified by PCR to generate products corresponding to messenger RNA (mRNA) encoding human gene products for GAPDH, RANKL, OPG and TRAIL (Table 1). Aliquots of PCR products were fractionated on 1% agarose gels stained with ethidium bromide. Gel pictures and quantification of signals were obtained after scanning with AlphaImager 2200 (Alpha Innotech Corporation, USA) and ImageJ software analysis (public domain Java image processing program). Messenger RNA expression of GAPDH, RANKL, OPG and TRAIL was also determined in bone stromal cells cultured from explants of cancellous bone derived from the femoral head as previously described (Gundle and Beresford, 1995). Cultures were passaged at least twice before the cells were harvested for RT–PCR.

### Melanoma-derived fibroblasts: effect on osteoclast formation

To determine whether melanoma-derived fibroblasts produce a soluble factor capable of inducing osteoclast formation, human PBMCs were cultured for 21 days in the presence and absence of melanoma-derived fibroblast CM. A dose–response curve (0–10%) established that 10% CM provided the optimal concentration for osteoclast formation. Subsequent experiments were set up as follows:

1. M-CSF (25 ng ml^−1^) (negative control).
2. M-CSF (25 ng ml^−1^), RANKL (30 ng ml^−1^) (positive control).
3. 10% CM, M-CSF (25 ng ml^−1^).
4. 10% CM, M-CSF (25 ng ml^−1^), ±OPG (500 ng ml^−1^) or RANK:Fc (500 ng ml^−1^).
5. 10% CM, M-CSF (25 ng ml^−1^), ±anti-human TNF-*α* antibody (10 *μ*g ml^−1^).

Cultures on coverslips and dentine slices were maintained for 14 and 21 days, respectively, with culture medium, CM and cytokines replenished every 3–4 days.

### *SK-Mel-29* melanoma cells: effect on osteoclast formation

*SK-Mel-29* cells were cultured in 25 cm^2^ culture flasks until confluent; CM was then extracted after further incubation of 48 h. A dose–response curve (0–10%) established that 10% CM provided the optimal concentration for osteoclast formation (Figure 6). Subsequent experiments with human PBMCs were set up as follows:

1. M-CSF (25 ng ml^−1^) (negative control).
2. M-CSF (25 ng ml^−1^), RANKL (30 ng ml^−1^) (positive control).
3. M-CSF (25 ng ml^−1^), 10% CM.
4. M-CSF (25 ng ml^−1^), 10% CM, ±OPG (500 ng ml^−1^) or RANK:Fc (500 ng ml^−1^).
5. M-CSF (25 ng ml^−1^), 10% CM, ±one of the following: anti-human TNF-*α* antibody (10 *μ*g ml^−1^); anti-human TGF-*β* antibody (500 ng ml^−1^); anti-human gp130 antibody (5 *μ*g ml^−1^); anti-human IL-8 antibody (10 *μ*g ml^−1^).

Peripheral blood mononuclear cell cultures on coverslips and dentine slices were maintained for 14 and 21 days, respectively, with culture medium and factors being replenished every 3–4 days.

### *SK-Mel-29* melanoma cells: total RNA extraction and RT–PCR

*SK-Mel-29* cells from 25 cm^2^ culture flasks were removed for RNA extraction by treatment with trypsin and the same protocol for RT–PCR and fractionation on agarose gels described above was used.

### *SK-Mel-29* melanoma cells: determining the nature of active moiety within CM

In order to provide information on the nature of the active moiety present in the *SK-Mel-29* CM, the CM was concentrated and the protein solute separated from low molecular weight compounds using an Amicon® Ultra PL-10, 10 000 nominal molecular weight limit (NMWL) centrifugal filter device (Millipore Corp., USA). The concentrated retentate (⩾10 kDa) was diluted in MEM and the ultrafiltrate (<10 kDa) was added neat to human PBMC cultures supplemented with M-CSF (25 ng ml^−1^). Cultures on coverslips and dentine slices were maintained for 14 and 21 days, respectively, with culture medium and factors replenished every 3–4 days.

### *SK-Mel-29* melanoma cells: effect on mature osteoclast activity

In order to determine if *SK-Mel-29* CM has any effect on mature osteoclast activity, osteoclastic giant cells from two specimens of fresh giant cell tumour of bone were isolated by digestion with collagenase as previously described (Lau *et al*, 2005) and cultured in the presence and absence of *SK-Mel-29* CM. Cultures on coverslips and dentine slices were maintained for 48 h.

### Statistical analysis

The extent of lacunar resorption was measured using an image analysis software (Adobe Photoshop, USA) as previously described (Quinn *et al*, 1998) and expressed as the mean percentage of surface area resorbed±standard error of mean (s.e.m.). In order to minimise the effect of batch-to-batch variation of PBMCs, all resorption data were normalised and expressed relative to the response obtained in PBMC cultures incubated with 25 ng ml^−1^ M-CSF and 30 ng ml^−1^ RANKL (positive control). Statistical significance was determined using the unpaired *t*-test and *P*-values <0.05 were considered significant.

## RESULTS

### Characterisation of TAMs isolated from melanoma

After incubation for 24 h on glass coverslips, the cells isolated from the metastatic melanomas, when cultured in the presence and absence of M-CSF and RANKL, strongly expressed the monocyte/macrophage marker CD14, which is not expressed by osteoclasts. These cells were negative for the osteoclast markers, TRAP and VNR, and the melanoma markers, HMB-45 and S100. Twenty-four hours culture of these cells on dentine slices, both in the presence and absence of M-CSF and RANKL, showed no evidence of lacunar resorption. The melanoma-derived mononuclear cells in long-term cultures thus expressed the phenotypic markers of macrophages and not osteoclasts or tumour cells.

### TAM–osteoclast differentiation

**Figure 1:**
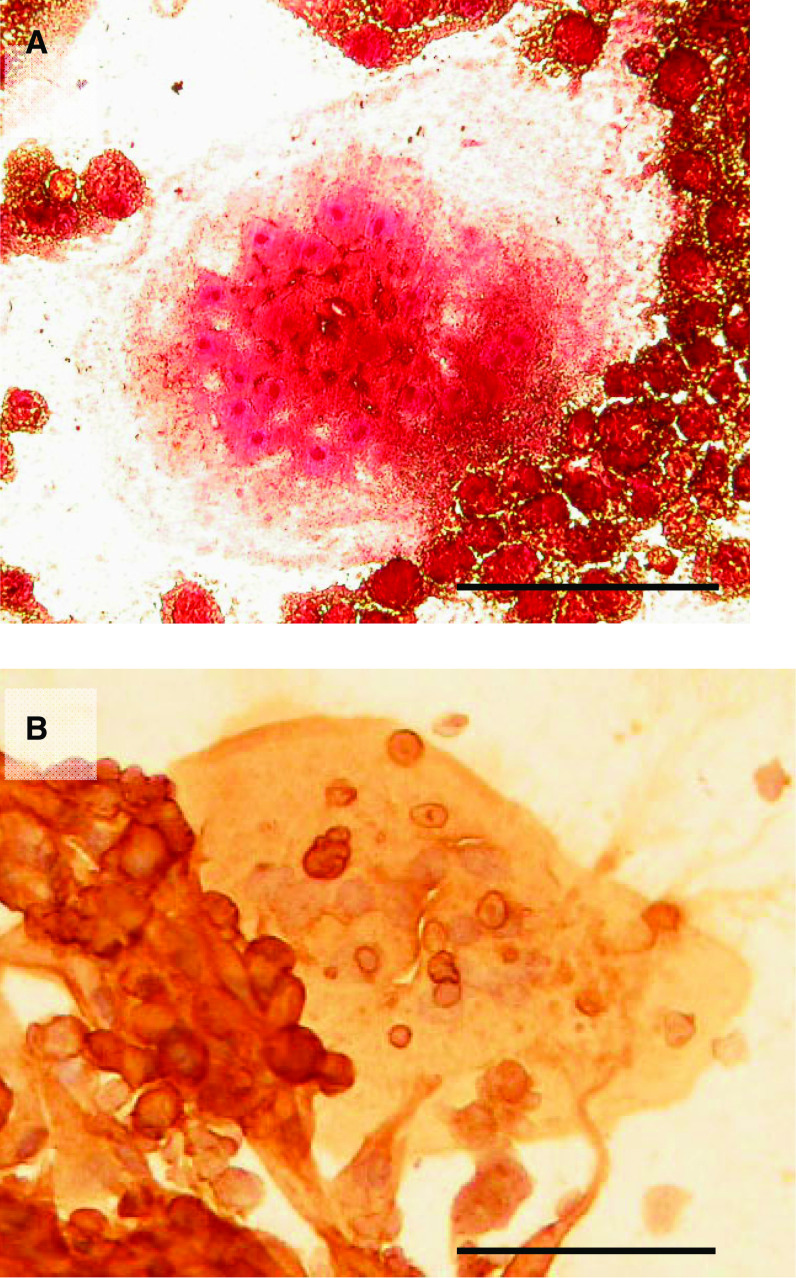
(**A**) TRAP and (**B**) VNR expression by multinucleated cells formed in 14-day melanoma TAM cultures in the presence of M-CSF and RANKL. Bar=500 *μ*m.

In the presence of RANKL and M-CSF, numerous multinucleated cells expressing the osteoclast-associated markers, TRAP and VNR, were found in 14-day TAM cultures incubated on glass coverslips (Figure 1). No expression of TRAP or VNR was seen when M-CSF or RANKL was omitted.

**Figure 2:**
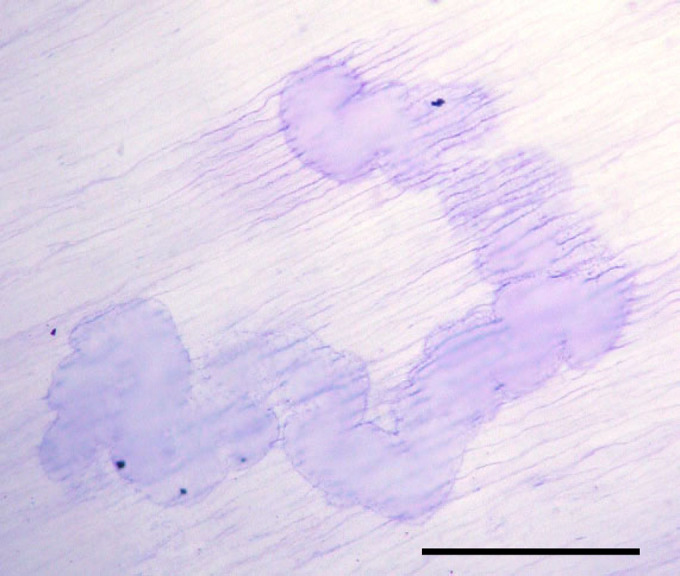
Large, compound lacunar resorption pit formed on a dentine slice in a 21-day melanoma TAM culture in the presence of M-CSF and RANKL (Toluidine blue staining). Bar=500 *μ*m.

After incubation for 21 days, the TAMs incubated on dentine slices, in the presence of M-CSF and RANKL, showed functional evidence of osteoclast differentiation with the formation of numerous areas of lacunar resorption; these were largely in the form of single large resorption pits or multiple compound areas of lacunar excavation on the dentine surface (Figure 2). In the absence of M-CSF or soluble RANKL, osteoclast formation and lacunar resorption was not seen.

**Figure 3:**
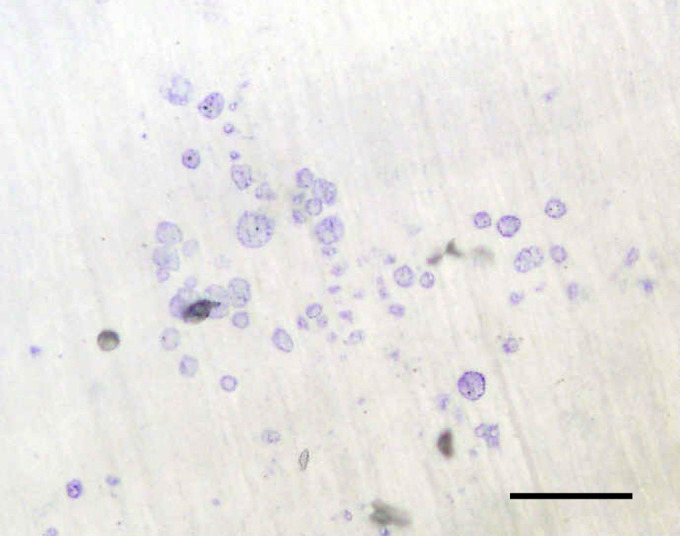
Small, single lacunar resorption pits formed on a dentine slice in a 21-day melanoma TAM culture in the presence of M-CSF, TNF-*α* and IL-1 (Toluidine blue staining). Bar=400 *μ*m.

Tumour-associated macrophages cultured with M-CSF, TNF-*α* and IL-1*α* also showed formation of TRAP+ and VNR+ multinucleated cells. Functional evidence of osteoclast formation under these conditions was also noted in 21-day cultures on dentine slices where a few small single round or ovoid resorption pits were formed (Figure 3).

### Effect of melanoma-derived fibroblasts on osteoclast formation

**Figure 4:**
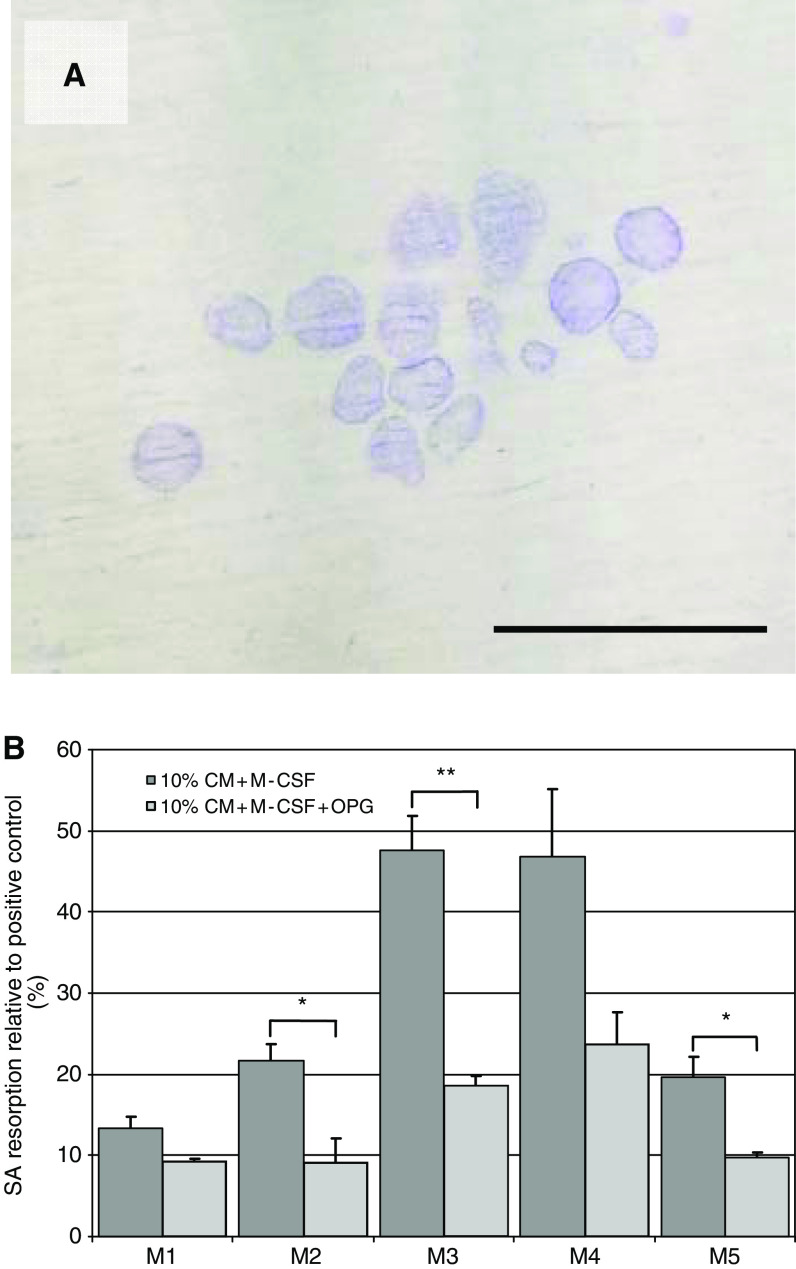
(**A**) Lacunar resorption pits formed on a dentine slice in a 21-day human PBMC culture in the presence of 10% melanoma-derived fibroblast conditioned medium (CM) (Toluidine blue staining). Bar=250 *μ*m. (**B**) Effect of 10% melanoma-derived fibroblast CM from five patients (M1–M5) on human monocyte–osteoclast differentiation. The data represent the mean % surface area (SA) lacunar resorption relative to the positive control (PBMC cultures with M-CSF and RANKL), in the absence and presence of OPG (500 ng ml^−1^). Error bars denote s.e.m. (*n*=3). ^*^P=0.02, ^**^P=0.002.

After three passages, the cells isolated from the melanomas by collagenase digestion consisted almost entirely of spindle-shaped, fibroblastic mononuclear cells, which did not stain for CD14, CD68, VNR, TRAP, HMB-45 and S100. No multinucleated cells were present in these cultures. Human PBMCs incubated with M-CSF and CM derived from these fibroblast cultures showed evidence of osteoclast formation with formation of TRAP+ and VNR+ multinucleated cells and evidence of lacunar resorption pit formation (Figure 4A). The addition of OPG to these cultures resulted in a marked decrease in the extent of lacunar resorption in all cases; this inhibition was statistically significant in three cases (Figure 4B). The addition of RANK:Fc to these cultures similarly inhibited osteoclast formation and resorption.

**Figure 5:**
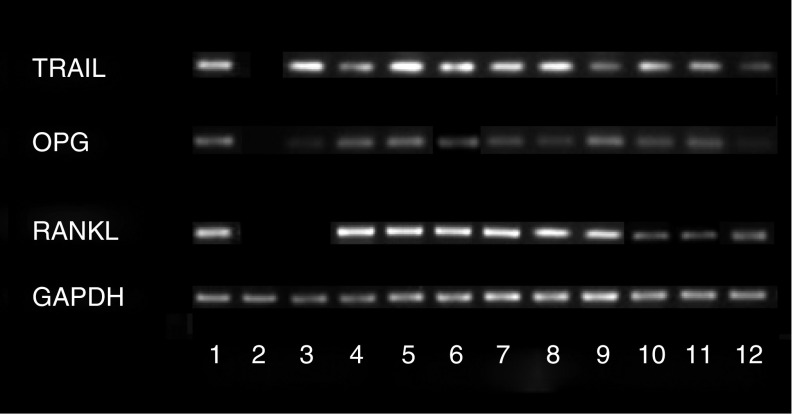
Expression of RANKL, OPG and TRAIL mRNA by fibroblasts derived from melanoma. Reverse transcription–polymerase chain reaction products were fractionated on agarose gel. Lane 1, positive control; lane 2, negative control; lane 3, *SK-Mel-29*; lanes 4–9, melanoma fibroblasts from 6 patients; lanes 10–12, normal bone stromal cells.

Using a semiquantitative RT–PCR method, signals generated by mRNA levels of RANKL, OPG and TRAIL were quantified relative to GAPDH. Expression of RANKL, OPG and TRAIL was noted in fibroblasts cultured from all melanoma specimens and was also noted in control bone stromal cells (Figure 5).

### Effect of *SK-Mel-29* melanoma cells on osteoclast formation

**Figure 6:**
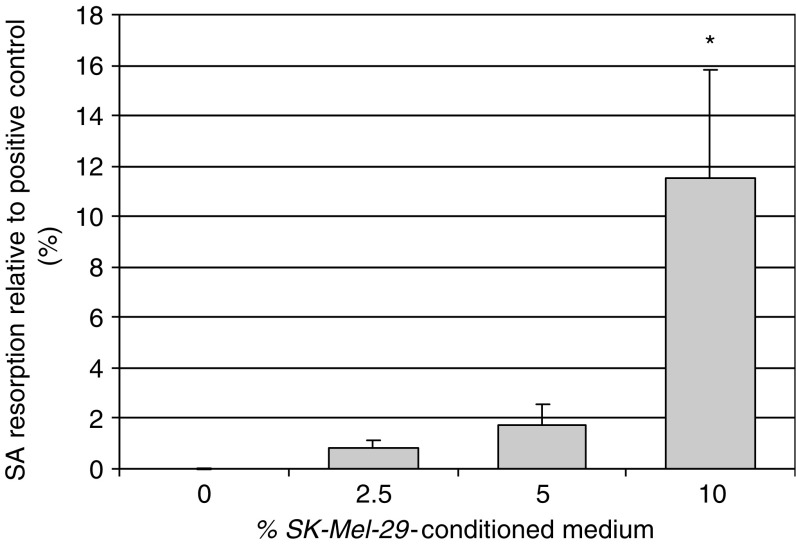
The effect of *SK-Mel-29* conditioned medium (CM) on human monocyte–osteoclast differentiation. The data represent the mean % surface area (SA) lacunar resorption relative to the positive control (PBMC cultures with M-CSF and RANKL). Error bars denote s.e.m. (*n*=6). ^*^ denotes the significant difference in lacunar resorption in PBMC cultures incubated in the absence and presence of 10% *SK-Mel-29* CM (*P*<0.01) relative to untreated control.

**Figure 7:**
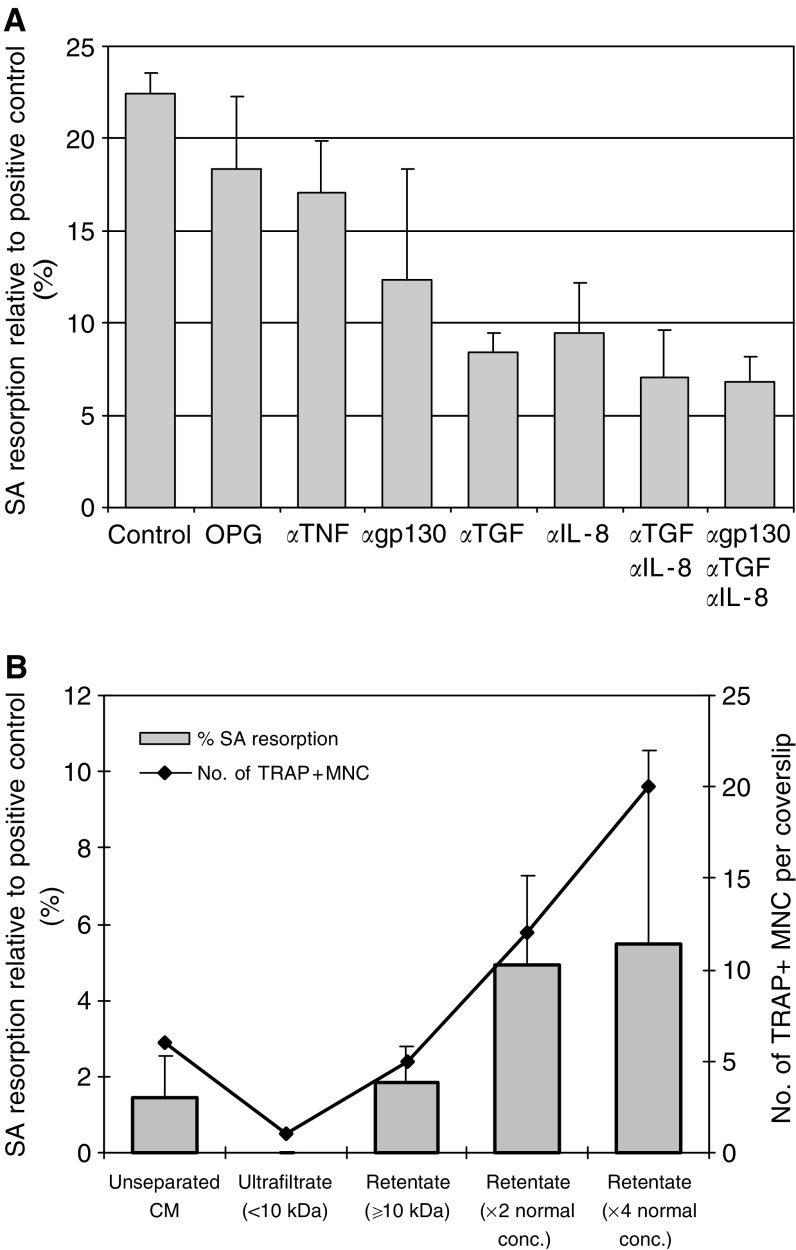
(**A**) The effect of OPG and neutralising antibodies to osteoclastogenic humoral factors on resorption in PBMC cultures incubated with M-CSF and 10% *SK-Mel-29* conditioned medium (CM). The data represent the mean % surface area (SA) lacunar resorption relative to the positive control (PBMC cultures with M-CSF and RANKL). Error bars denote s.e.m. (*n*=6). (**B**) The effect of the low and high molecular weight components within *SK-Mel-29* CM on human monocyte–osteoclast differentiation. The data represent the mean % surface area (SA) lacunar resorption relative to the positive control (PBMC cultures with M-CSF and RANKL). Error bars denote s.e.m. (*n*=6). In addition, the mean number of TRAP+ multinucleated cells (MNC) is also demonstrated (*n*=2).

Cultures of human PBMCs incubated with M-CSF and *SK-Mel-29* CM resulted in the formation of TRAP+ and VNR+ multinucleated cells capable of lacunar resorption. The amount of lacunar resorption was dose-dependent, with maximal resorption seen at CM concentration of 10% (Figure 6). The addition of OPG or RANK:Fc to *SK-Mel-29* CM-treated PBMC cultures did not abolish osteoclast formation or significantly inhibit lacunar resorption, suggesting that *SK-Mel-29* CM stimulated osteoclastogenesis via a RANKL-independent mechanism. However, the addition to *SK-Mel-29* CM-treated PBMC cultures of neutralising antibodies to humoral factors known to stimulate osteoclasto4genesis, specifically antibodies to the receptors for IL-6/-11 (gp130) and TGF-*β*, and antibodies to the cytokines TNF-*α* and IL-8, also failed to abolish osteoclast formation or significantly inhibit resorption (Figure 7A).

Cultures of human PBMCs incubated in the presence of M-CSF and the high molecular weight (⩾10 kDa) *SK-Mel-29* CM retentate, separated using the centrifugal filter device, resulted in the formation of a small number of TRAP+ multinucleated cells capable of lacunar resorption. The lacunar resorption pits produced were similar in appearance to those formed when unseparated CM was added to PBMC cultures. In PBMC cultures to which *SK-Mel-29* CM ultrafiltrate (<10 kDa) was added, TRAP+ multinucleated cells and lacunar resorption was not seen. This confirms that the osteoclastogenic activity of the *SK-Mel-29* CM is unique and that the active moiety has a molecular weight ⩾10 kDa. Further confirmation of the active moiety present in the retentate was seen in cultures of human PBMCs and increasing concentrations of *SK-Mel-29* CM retentate. A dose-dependent response was seen, with a proportional increase in the formation of TRAP+ multinucleated cells and lacunar resorption at higher concentrations of *SK-Mel-29* CM retentate (Figure 7B).

RT–PCR studies showed that *SK-Mel-29* cells expressed the mRNA for OPG and TRAIL but not RANKL (Figure 5).

### Effect of *SK-Mel-29* cells on mature osteoclast activity

Giant cell tumour of bone is characterised morphologically by the presence of numerous giant cells, which express the cytochemical and functional phenotypic characteristics of osteoclasts. This effect was evident in cultures with no added factors, where numerous TRAP+ and VNR+ multinucleated cells were seen and there was extensive lacunar excavation of the dentine surface. The addition of *SK-Mel-29* CM did not significantly influence the resorption activity of the osteoclasts isolated from the giant cell tumour.

## DISCUSSION

Bone metastases in melanoma are commonly osteolytic and associated with marked osteoclastic resorption. In this study, we have identified a number of cellular and humoral factors that contribute to osteoclast formation in metastatic melanoma. First, we have shown that TAMs in metastatic melanomas have the ability to differentiate into functional mature osteoclasts. This osteoclast formation is mediated mainly by a RANKL-dependent mechanism, but TNF-*α* (and possibly other cytokines/growth factors) is also capable of inducing osteoclast formation. We also found that melanoma-derived fibroblasts, like bone stromal cells, express RANKL mRNA and that these cells support osteoclast formation by a RANKL-dependent mechanism. In contrast, melanoma cells produce a soluble factor that appears to promote osteoclast formation by a mechanism independent of RANKL.

Melanoma cells are known to secrete several factors, which induce the recruitment of TAMs. These include monocyte chemotactic protein-1, M-CSF, GM-CSF and VEGF-C (Skobe *et al*, 2001). Tumour-associated macrophages form a major component of the inflammatory cell infiltrate within and around primary and metastatic melanomas, and TAM numbers have been shown to correlate with melanoma invasion (Torisu *et al*, 2000). These TAMs produce cytokines and growth factors, including IL-8, VEGF and FGF, which stimulate tumour growth and angiogenesis in melanomas (Torisu *et al*, 2000; Nesbit *et al*, 2001; Shimizu *et al*, 2001; Callejo *et al*, 2004). We have previously shown that mouse and human TAMs isolated from primary and metastatic breast carcinomas are capable of differentiation into TRAP+ osteoclastic cells capable of extensive lacunar resorption when these cells are co-cultured with bone-derived stromal cells and M-CSF (Quinn *et al*, 1992, 1994). In this study, we found that melanoma-derived TAMs are also capable of osteoclast differentiation by a RANKL-dependent mechanism. Tumour-associated macrophages isolated from secondary melanomas expressed the monocyte/macrophage marker CD14 and were negative for the osteoclast markers TRAP and VNR and incapable of carrying out lacunar resorption. However, following treatment with RANKL and M-CSF, these cells differentiated into TRAP^+^ and VNR^+^ multinucleated cells capable of extensive lacunar resorption.

Tumor necrosis factor-*α* and other cytokines/growth factors that induce osteoclast formation by a RANKL-independent mechanism, are known to be produced by melanoma cells (Lazar-Molnar *et al*, 2000). We found that melanoma TAMs were also capable of osteoclast differentiation when cultured in the presence of M-CSF, TNF-*α* and IL-1. In contrast to the numerous large multinucleated osteoclasts and extensive areas of lacunar resorption produced when TAMs were cultured with RANKL, osteoclasts formed in the presence of TNF-*α* and IL-1 were much smaller and contained fewer than four nuclei; resorption areas produced by these osteoclasts were also correspondingly smaller, being mainly in the form of single resorption pits. We have previously demonstrated that this is characteristic of TNF-*α* and other cytokine-induced mechanisms of osteoclast differentiation (Kudo *et al*, 2002).

Melanoma–stromal cell interactions are known to play an important role in tumour growth and metastasis. Melanoma cells are known to produce M-CSF *in vitro* (Perez *et al*, 2001, Varney *et al*, 2005). As some fibroblast populations are known to express RANKL and OPG, we investigated whether fibroblasts derived from secondary melanomas expressed these osteoclastogenic factors and were capable of supporting osteoclast formation. We found that melanoma fibroblasts express RANKL, OPG and TRAIL; the latter is known to bind OPG. We also found that the addition of CM derived from cultures of these melanoma fibroblasts could support monocyte–osteoclast differentiation and that this process was markedly inhibited by the addition of OPG. Our findings suggest that melanoma fibroblasts may induce osteoclast formation by a RANKL-dependent mechanism, and that this most likely occurs through the release of soluble RANKL. This feature has been reported in fibroblasts found in giant cell tumour of bone (Lau *et al*, 2005), a tumour that contains numerous osteoclasts, but not in normal fibroblast populations, including skin, bone marrow stromal cells, as well as fibroblasts derived from other non-neoplastic tissue (Quinn *et al*, 2000; Sabokbar *et al*, 2005; Sun *et al*, 2006). Stromal cells in primary melanoma are known to stimulate tumour progression and these cells may be activated in bone metastases to stimulate osteoclast differentiation and bone resorption (Spatz *et al*, 2002).

Melanoma cells have previously been shown to stimulate the recruitment and activation of osteoclasts that carry out malignant bone resorption (Clohisy *et al*, 1996). Melanoma cells release a number of osteoclastogenic factors including TGF-*β*, IL-6, M-CSF, GM-CSF and TNF-*α* (Perez *et al*, 2001); these cells also upregulate osteoblast expression of factors known to stimulate RANKL-dependent osteoclastogenesis. Melanoma cells, however, have not been shown to express RANKL (Chikatsu *et al*, 2000), and we did not identify RANKL mRNA in *SK-Mel-29* human melanoma cells. This is in keeping with our finding that neither OPG nor RANK:Fc inhibited monocyte–osteoclast differentiation induced by the addition of melanoma cell CM. The osteoclastogenesis induced by the soluble factor produced by *SK-Mel-29* cells thus appeared to be RANKL-independent. The molecular weight of this soluble factor was ⩾10 kDa as only the retentate of the CM from the *SK-Mel-29* cells induced osteoclastic formation. Attempts to block all known humoral factors that have been shown to stimulate RANKL-independent osteoclast formation by the addition of neutralising antibodies to TNF-*α*, IL-6/11, IL-8 and TGF-*β* alone inhibited osteoclast formation. Melanoma cells are known to produce a number of cytokines and growth factors which have been implicated in osteoclastogenesis, and it is thus possible that more than one humoral factor produced by *SK-Mel-29* cells may contribute to the induction of osteoclast formation.

In summary, we have shown that the major cellular components of a metastatic melanoma play an important role in promoting the osteoclast formation that is required for tumour osteolysis. TRAP+ osteoclasts can be formed from TAMs in melanoma metastases and this process appears to occur by both RANKL-dependent and RANKL-independent mechanisms. The former is likely to involve an interaction with bone stromal cells and tumour fibroblasts, both of which express RANKL. Melanoma cells also produce a soluble factor that stimulates osteoclast formation by a RANKL-independent mechanism. In early skeletal metastases where a relatively high macrophage: tumour cell ratio and large number of osteoclasts are typically found (Galasko, 1976; Bugelski *et al*, 1985), RANKL-dependent and RANKL-independent mechanism of pathological bone resorption are likely to play a role in the establishment of a metastatic malignant melanoma in bone.

## Figures and Tables

**Table 1:** Human primer sequences used in amplification

	**Primer sequence**	**Size of product (bp)**	**Annealing temp. (°C)**
GAPDH	Forward 5′-CAC TGA CAC GTT GGC AGT GG-3′	360	60
	Reverse 5′-CAT GGA GAA GGC TGG GGC TC-3′		
OPG	Forward 5′-ATG AAC AAG TTG CTG TGC TG-3′	354	58
	Reverse 5′-GCA GAA CTC TAT CTC AAG GTA-3′		
RANKL	Forward 5′-CAG ATG GAT CCT AAT AGA AT-3′	324	56
	Reverse 5′-ATG GGA ACC AGA TGG GAT GTC-3′		
TRAIL	Forward 5′-ATC ATG GCT ATG ATG GAG GT-3′	315	58
	Reverse 5′-AAC TGT AGA AAT GGT TTC CTC-3′		
